# Interaction analysis of high-risk pathological features on adjuvant chemotherapy survival benefit in stage II colon cancer patients: a multi-center, retrospective study

**DOI:** 10.1186/s12885-023-11196-4

**Published:** 2023-09-18

**Authors:** Kexuan Li, Fuqiang Zhao, Yuchen Guo, Qingbin Wu, Shuangling Luo, Junling Zhang, Heli Li, Shidong Hu, Bin Wu, Guole Lin, Huizhong Qiu, Beizhan Niu, Xiyu Sun, Lai Xu, Junyang Lu, Xiaohui Du, Zheng Wang, Xin Wang, Liang Kang, Ziqiang Wang, Quan Wang, Qian Liu, Yi Xiao

**Affiliations:** 1 Division of Colorectal Surgery, Department of General Surgery, Peking Union Medical College Hospital, Chinese Academy of Medical Sciences and Peking Union Medical College, No.1 Shuai Fu Yuan Road, Dongcheng District, Beijing, 100730 China; 2 Department of Colorectal Surgery, National Cancer Center/National Clinical Research Center for Cancer/Cancer Hospital, Chinese Academy of Medical Sciences and Peking Union Medical College, No.17 Panjiayuan Nanli, Chaoyang District, Beijing, 100021 China; 3 Department of Gastrointestinal Surgery, General Surgery Center, The First Hospital of Jilin University, No.1 Xin Min Avenue, Chaoyang District, Changchun, 130021 China; 4 Colorectal Cancer Center, Department of General Surgery, West China Hospital, Sichuan University, No.37 Guo Xue Xiang, Wuhou District, Chengdu, 610041 China; 5 Department of General Surgery (Colorectal Surgery), The Sixth Affiliated Hospital, Sun Yat-sen University, No.26 Yuan Cun Er Heng Road, Tianhe District, Guangzhou, 510655 China; 6 Guangdong Provincial Key Laboratory of Colorectal and Pelvic Floor Diseases, The Sixth Affiliated Hospital, Sun Yat-sen University, No.26 Yuan Cun Er Heng Road, Tianhe District, Guangzhou, 510655 China; 7 Biomedical Innovation Center, The Sixth Affiliated Hospital, Sun Yat-sen University, No.26 Yuan Cun Er Heng Road, Tianhe District, Guangzhou, 510655 China; 8 Department of Gastrointestinal Surgery, Peking University First Hospital, No.8 Xi Shi Ku Street, Xicheng District, Beijing, 100034 China; 9 Department of Gastrointestinal Surgery, Union Hospital, Tongji Medical College, Huazhong University of Science and Technology, No.1277 Jiefang Avenue, Wuhan, 430022 China; 10 Department of General Surgery, The First Medical Center, Chinese People’s Liberation Army (PLA) General Hospital, No.28 Fu Xing Road, Haidian District, Beijing, 100853 China

**Keywords:** Colon cancer, High-risk pathological features, Adjuvant chemotherapy

## Abstract

**Background:**

We aimed to analyze the benefit of adjuvant chemotherapy in high-risk stage II colon cancer patients and the impact of high-risk factors on the prognostic effect of adjuvant chemotherapy.

**Methods:**

This study is a multi-center, retrospective study, A total of 931 patients with stage II colon cancer who underwent curative surgery in 8 tertiary hospitals in China between 2016 and 2017 were enrolled in the study. Cox proportional hazard model was used to assess the risk factors of disease-free survival (DFS) and overall survival (OS) and to test the multiplicative interaction of pathological factors and adjuvant chemotherapy (ACT). The additive interaction was presented using the relative excess risk due to interaction (RERI). The Subpopulation Treatment Effect Pattern Plot (STEPP) was utilized to assess the interaction of continuous variables on the ACT effect.

**Results:**

A total of 931 stage II colon cancer patients were enrolled in this study, the median age was 63 years old (interquartile range: 54–72 years) and 565 (60.7%) patients were male. Younger patients (median age, 58 years vs 65 years; *P* < 0.001) and patients with the following high-risk features, such as T4 tumors (30.8% vs 7.8%; *P* < 0.001), grade 3 lesions (36.0% vs 22.7%; *P* < 0.001), lymphovascular invasion (22.1% vs 6.8%; *P* < 0.001) and perineural invasion (19.4% vs 13.6%; *P* = 0.031) were more likely to receive ACT. Patients with perineural invasion showed a worse OS and marginally worse DFS (hazardous ratio [HR] 2.166, 95% confidence interval [CI] 1.282–3.660, *P* = 0.004; HR 1.583, 95% CI 0.985–2.545, *P* = 0.058, respectively). Computing the interaction on a multiplicative and additive scale revealed that there was a significant interaction between PNI and ACT in terms of DFS (HR for multiplicative interaction 0.196, *p* = 0.038; RERI, -1.996; 95%CI, -3.600 to -0.392) and OS (HR for multiplicative interaction 0.112, *p* = 0.042; RERI, -2.842; 95%CI, -4.959 to -0.725).

**Conclusions:**

Perineural invasion had prognostic value, and it could also influence the effect of ACT after curative surgery. However, other high-risk features showed no implication of efficacy for ACT in our study.

**Trial registration:**

This study is registered on ClinicalTrials.gov, NCT03794193 (04/01/2019).

**Supplementary Information:**

The online version contains supplementary material available at 10.1186/s12885-023-11196-4.

## Background

The benefit of adjuvant chemotherapy (ACT) for stage III colon cancer patients has been well established from existing literatures [1–3]. However, the topic of whether patients with stage II colon cancer would benefit from ACT following radical surgical resection is still in controversy. A pooled analysis of 12 meta-analyses and 37 randomized controlled trials presented in the 2004 ASCO guideline suggested that ACT would not help stage II colon cancer patients [4]. As for recent RCTs, the SACURA trial [5] demonstrated no significant differences in 5-year DFS, relapse-free survival (RFS), or OS between the surgery-only group and the Tegafur/uracil oral intake group. Considering the heterogeneity among stage II patients, determining a specific subgroup which might benefit from chemotherapy came to the focus of researchers. The National Comprehensive Cancer Network (NCCN) guideline [6] suggests patients with the following high-risk pathological features receive ACT after curative resection: T4 tumors, poorly differentiated/undifferentiated histology, lymphatic/vascular invasion, bowel obstruction, < 12 lymph nodes examined, perineural invasion, localized perforation, tumor budding, or close, indeterminate, positive margins. However, no high-quality evidence has indicated risk features and corresponding chemotherapy selection, thus observation is still optional according to the guideline [4, 6].

Efforts made into the treatment effect of ACT in high-risk patients have demonstrated contradictory findings in several studies [7–9], but few have looked into the specific high-risk feature subgroups, and no previous studies have analyzed the interaction between certain high-risk features and chemotherapy effect on survival to the author’s knowledge. In our study, aiming to explore the effect modification power of high-risk pathological features, we tested the multiplicative as well as the additive interaction and implemented subpopulation treatment effect pattern plot (STEPP) analysis to further determine an optimal high-risk subgroup, significant interaction was observed depending on PNI status.

## Materials and methods

### Study design and participants

This study was a multi-center, retrospective, and observational study, which collected data from the following eight centers: Peking Union Medical College Hospital, National Cancer Center/National Clinical Research Center for Cancer/Cancer Hospital, The First Hospital of Jilin University, Peking University First Hospital, West China Hospital of Sichuan University, The 6th Affiliated Hospital of Sun Yat-sen University, Union Hospital affiliated to Tongji Medical College, Chinese People’s Liberation Army (PLA) General Hospital from January 1, 2016, to December 31, 2017. This study was approved by the Ethics Committee of Peking Union Medical College Hospital (ZS-2888). Approval of the local ethics committee at each center was also obtained. This study is registered on ClinicalTrials.gov, NCT03794193 (04/01/2019).

Eligible participants were selected according to the following criteria. For inclusion, the patients are supposed to be: (1) stage II colon adenocarcinoma, which was pathologically diagnosed as pT_3-4_N_0_ colon adenocarcinoma and no distant metastasis was found before and during surgery; (2) diagnosed with a tumor located from cecum to sigmoid colon. The patients are excluded if they (1) are diagnosed with multiple primary colorectal cancer; (2) have a history of other malignant neoplasms; (3) were treated with neoadjuvant therapy; (4) were treated with palliative surgery.

For high-risk stage II patients, a 6-month postoperative ACT administered with fluoropyrimidine-based agents with/without Oxaliplatin, including capecitabine or tegafur single-agent, XELOX or FOLFOX regimens was considered the standard treatment.

### Variables and outcomes

The following variables were obtained from the patient's medical record: gender, age, American Society of Anesthesiologists (ASA) classification, ACT administration, and the pathological features included in this study were: T-stage, lymphovascular invasion (LVI), perineural invasion (PNI), number of lymph nodes examined, poorly differentiated or undifferentiated adenocarcinoma, mucinous adenocarcinoma, signet ring cell carcinoma and circumferential resection margin (CRM). Perineural invasion is defined by the encirclement of at least one third of a nerve's circumference by cancer cells, which can be found in any of the three nerve layers—the epineurium, perineurium, and endoneurium [10]. Since there’s no differentiation information of mucinous adenocarcinoma and signet ring cell adenocarcinoma in certain centers, we incorporated poorly differentiated/undifferentiated adenocarcinoma, mucinous adenocarcinoma or signet ring cell carcinoma into an integrated variable, i.e. grade 3 histology [11]. All pathological indicators were retrieved from the pathology reports stored in the hospital information system. The pathology reports were each primarily written by a junior pathologist and then reviewed by a senior pathologist based on the formalin-fixed and paraffin-embedded surgical specimen. The primary endpoint was disease-free survival of patients with colon cancer, which was defined as the time from the date of surgery to the first confirmed local recurrence, distant metastasis, or all-cause death, calculated in months. The secondary endpoint was overall survival (OS), which is defined as the time from the surgical date to all-cause death, calculated in months.

### Statistical analysis

In terms of sample size, the number of outcomes needs to be ten times the number of candidate predictors to meet the Events Per Variable (EPV) 10 criterion, and up to 7 predictors (4 adjusting terms + pathological features term + chemotherapy term + interaction term) were included in the analysis of this study, implying that at least 70 outcomes were required. In this study, a total of 100 patients reached the DFS endpoint, 71 patients reached the OS endpoint, meeting the sample size criteria. The missing values were imputed using the missForest package in R [12].

For those who followed a normal distribution, continuous variables were summarized as mean (SD), and analyzed by the t-test; those who didn’t were summarized as the median (IQR) and analyzed by Mann–Whitney U test. Categorical variables were presented as number (%) and analyzed by the χ2 test or Fisher’s exact test. In the subgroup analysis, Cox proportional hazard models were used to estimate hazard ratios (HRs) with 95% CI to evaluate the relationships of receipt of ACT with DFS and OS, stratified by specific high-risk features. To test for multiplicative interaction, the Cox models included a treatment term, a clinical-pathological term, and an interaction term. And to test for covariate-adjusted multiplicative interaction, terms of confounding factors (sex, age, ASA classification and PNI) were also added. The multiplicative interaction was presented as the HR and *p*-value of the interaction term. The additive interaction was assessed using the relative excess risk due to interaction (RERI), and Li’s [13] method of calculating interaction in proportional hazards models was adopted. A multiplicative interaction HR less than 1.0 or an additive interaction parameter less than 0 implies that individuals with specific pathological characteristics benefit more from ACT than those without.

The Subpopulation Treatment Effect Pattern Plot (STEPP) [14] is a graphical tool for estimating treatment effects for overlapping patient subpopulations defined by a covariate of interest. The resulting treatment hazards ratio estimates (surgery plus ACT versus surgery alone) of subpopulation by median age and lymph nodes harvest number are shown in a graphical manner. *P* < 0.05 was considered statistically significant. SPSS Statistics for Windows (version 25.0; Armonk, NY: IBM Corporation) and R software (version 4.1.2; R Core Team (2016), Vienna, Austria) were employed to conduct statistical analyses.

## Results

### Baseline characteristics

**Fig. 1:**
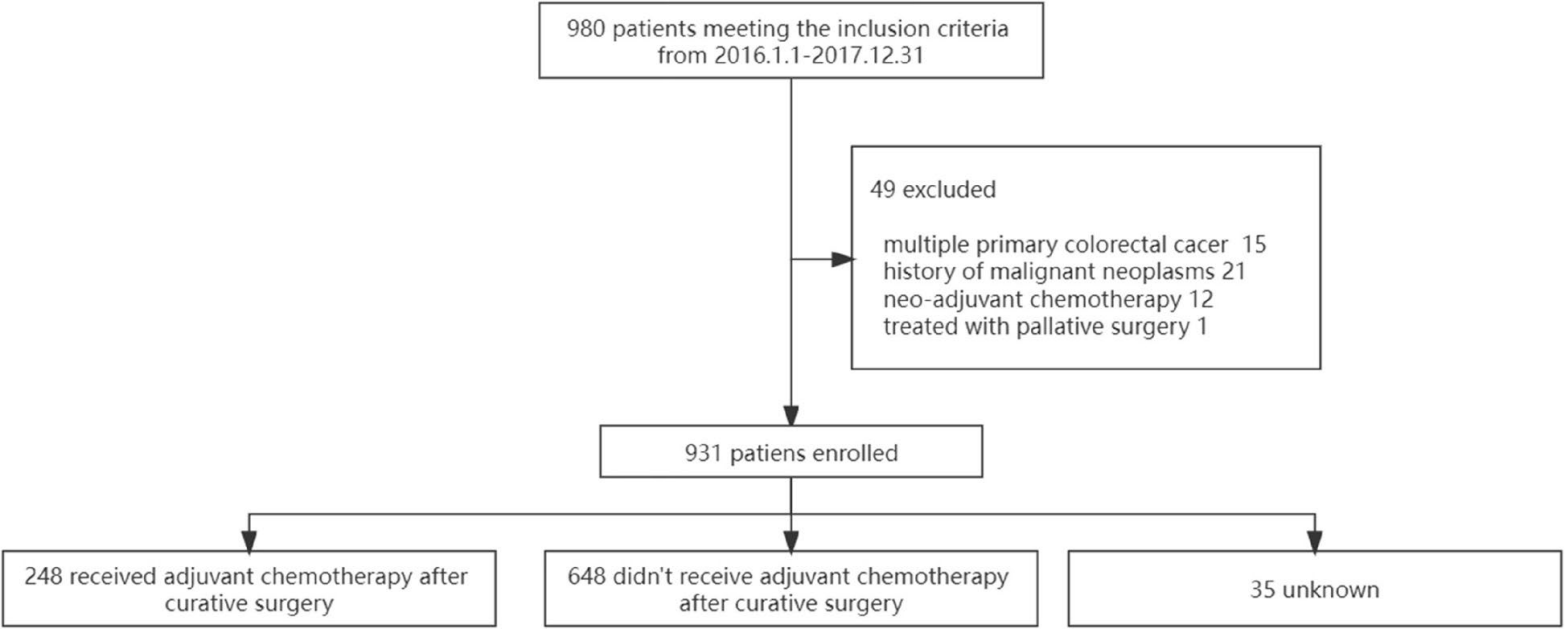
Flowchart of the selection process of patients

Of 931 stage II colon cancer patients enrolled (Fig. 1), 565 patients were male and 366 patients were female. The median age was 63 years old (IQR: 54–72 years). Patients with pathological T4 stage accounted for 14.1% of the population, whereas patients with lymph node harvest < 12, grade 3 tumor, LVI, PNI, CRM accounted for 6.7%, 26.3%, 10.8%, 14.8%, 0.7% respectively. 248 (26.6%) patients underwent adjuvant chemotherapy after curative surgery, whereas 648 (69.6%) didn’t (Table 1). Since some centers were unable to collect complete ACT regimens and duration of treatment, all patients who completed at least 1 cycle of ACT were included in the ACT group.

**Table 1:** Baseline Characteristics of Study Cohort (before imputation)

Clinical pathological features	Total	Percentage (%)
Sex		
Male	565	60.7
Female	366	39.3
Age	63.00 (54.00, 72.00)	
Missing	1	0.1
ASA classification		
I	154	16.5
II	650	69.8
III	120	12.9
IV	2	0.2
Missing	5	0.5
T stage		
T3	800	85.9
T4	131	14.1
Lymph node harvest < 12		
Yes	62	6.7
No	866	93.0
Missing	3	0.3
Histological grade		
G3	245	26.3
G1-2	685	73.6
Missing	1	0.1
LVI		
Yes	101	10.8
No	809	86.9
Missing	21	2.3
PNI		
Yes	138	14.8
No	765	82.2
Missing	28	3.0
CRM		
Yes	6	0.7
No	911	97.9
Missing	14	1.5
Adjuvant chemotherapy		
Yes	248	26.6
No	648	69.6
Missing	35	3.8

*ASA* American Society of Anesthesiologists, *LVI* Lymphovascular invasion, *PNI* Perineural invasion, *CRM* Circumferential resection margin

### Comparison of clinicopathological characteristic in patients who received ACT and those without

Patients who received ACT were younger (median age, 58 years vs 65 years; *P* < 0.001), have lower ASA classification (ASA III-IV: 8.7% vs 14.7%, *P* = 0.016), and tended to have the following pathological features, such as T4 tumors (30.8% vs 7.8%; *P* < 0.001), grade 3 lesions (36.0% vs 22.7%; *P* < 0.001), lymphovascular invasion (22.1% vs 6.8%; *P* < 0.001) and perineural invasion (19.4% vs 13.6%; *P* = 0.031). There was no significant difference in sex, number of lymph node harvest between patients who received ACT and those who did not (Table 2).

**Table 2:** Comparison of clinicopathological factors, stratified by adjuvant chemotherapy administration

	**ACT group** **% (*N* = 253)**	**Non-ACT group** **% (*N* = 678)**	*P*
Sex (Male)	163 (64.4)	402 (59.3)	0.175
Age (year)	58 (50, 66)	65 (56, 73)	< 0.001
ASA III/IV	22 (8.7)	100 (14.7)	0.016
T4	78 (30.8)	53 (7.8)	< 0.001
Lymph node harvest < 12	13 (5.1)	49 (7.2)	0.302
G3	91 (36.0)	154 (22.7)	< 0.001
LVI	56 (22.1)	46 (6.8)	< 0.001
PNI	49 (19.4)	92 (13.6)	0.031

*ACT* Adjuvant chemotherapy, *ASA* American Society of Anesthesiologists, *LVI* Lymphovascular invasion, *PNI* Perineural invasion

### Survival analysis

The median follow-up time was 50 months (IQR 44–57 months) in all patients. The DFS rate at 3-years was 90.5% and the OS rate at 3-years was 94.8%. In univariate analyses, elder patients, patients with higher ASA classification, and PNI had a significant inferior DFS compared with those who did not (HR 1.043, 95% CI 1.025–1.061, *P* < 0.001; HR 2.994, 95% CI 1.933–4.638, *P* < 0.001; HR 1.870, 95% CI 1.173–2.979, *P* = 0.008, respectively). Patients presenting with the features mentioned above also had a significant inferior OS (HR 1.067, 95% CI 1.043–1.090, *P* < 0.001; HR 3.567, 95% CI 2.152–5.911, *P* < 0.001; HR 2.464, 95% CI 1.469–4.135, *P* < 0.001, respectively). Variables detected by univariate analysis with a *p*-value < 0.2 were then entered into multivariate analyses. Sex, age, ASA classification were independent risk factors for DFS (HR 1.563, 95% CI 1.021–2.394, *P* = 0.040; HR 1.034, 95% CI 1.015–1.052, *P* < 0.001; HR 2.228, 95% CI 1.386–3.584, *P* < 0.001, respectively), and the prognostic effect of PNI on DFS reached borderline significance (HR 1.583, 95% CI 0.985–2.545, *P* = 0.058) (Table 3). Likewise, age, ASA classification and PNI were independent risk factors for OS (HR 1.054, 95% CI 1.029–1.078, *P* < 0.001; HR 2.135, 95% CI 1.242–3.668, *P* = 0.006; HR 2.166, 95% CI 1.282–3.660, *P* = 0.004, respectively) (Table 3).

**Table 3:** Univariate and multivariate association between clinicopathological features and survival outcomes

	DFS				OS			
	Univariate analysis		Multivariate analysis		Univariate analysis		Multivariate analysis	
	HR (95% CI)	*P*	HR (95% CI)	*P*	HR (95% CI)	*P*	HR (95% CI)	*P*
Sex (Male)	1.378 (0.905, 2.098)	0.135	1.563 (1.021, 2.394)	0.040	1.326 (0.807, 2.181)	0.266		
Age (year)	1.043 (1.025, 1.061)	< 0.001	1.034 (1.015, 1.052)	< 0.001	1.067 (1.043, 1.090)	< 0.001	1.054 (1.029, 1.078)	< 0.001
ASA III-IV	2.994 (1.933, 4.638)	< 0.001	2.228 (1.386, 3.584)	< 0.001	3.567 (2.152, 5.911)	< 0.001	2.135 (1.242, 3.668)	0.006
T4	1.607 (0.985, 2.624)	0.058	1.506 (0.915, 2.476)	0.107	1.154 (0.607, 2.195)	0.662		
Lymph node harvest < 12	1.160 (0.538, 2.620)	0.704			0.704 (0.221, 2.240)	0.553		
G3	0.876 (0.554, 1.386)	0.572			0.661 (0.369, 1.187)	0.166	0.725 (0.401, 1.312)	0.288
LVI	0.800 (0.404, 1.588)	0.524			0.606 (0.244, 1.504)	0.280		
PNI	1.870 (1.173, 2.979)	0.008	1.583 (0.985, 2.545)	0.058	2.464 (1.469, 4.135)	< 0.001	2.166 (1.282, 3.660)	0.004
ACT	0.775 (0.483, 1.244)	0.292			0.618 (0.339, 1.253)	0.117	0.936 (0.500, 1.752)	0.835

*DFS* Disease-free survival, *OS* Overall survival, *HR* Hazardous ratio, *ASA* American Society of Anesthesiologists, *LVI* Lymphovascular invasion, *PNI* Perineural invasion, *ACT* Adjuvant chemotherapy

### Interaction analysis comparing chemotherapy effect in different clinicopathological subgroups

**Fig. 2:**
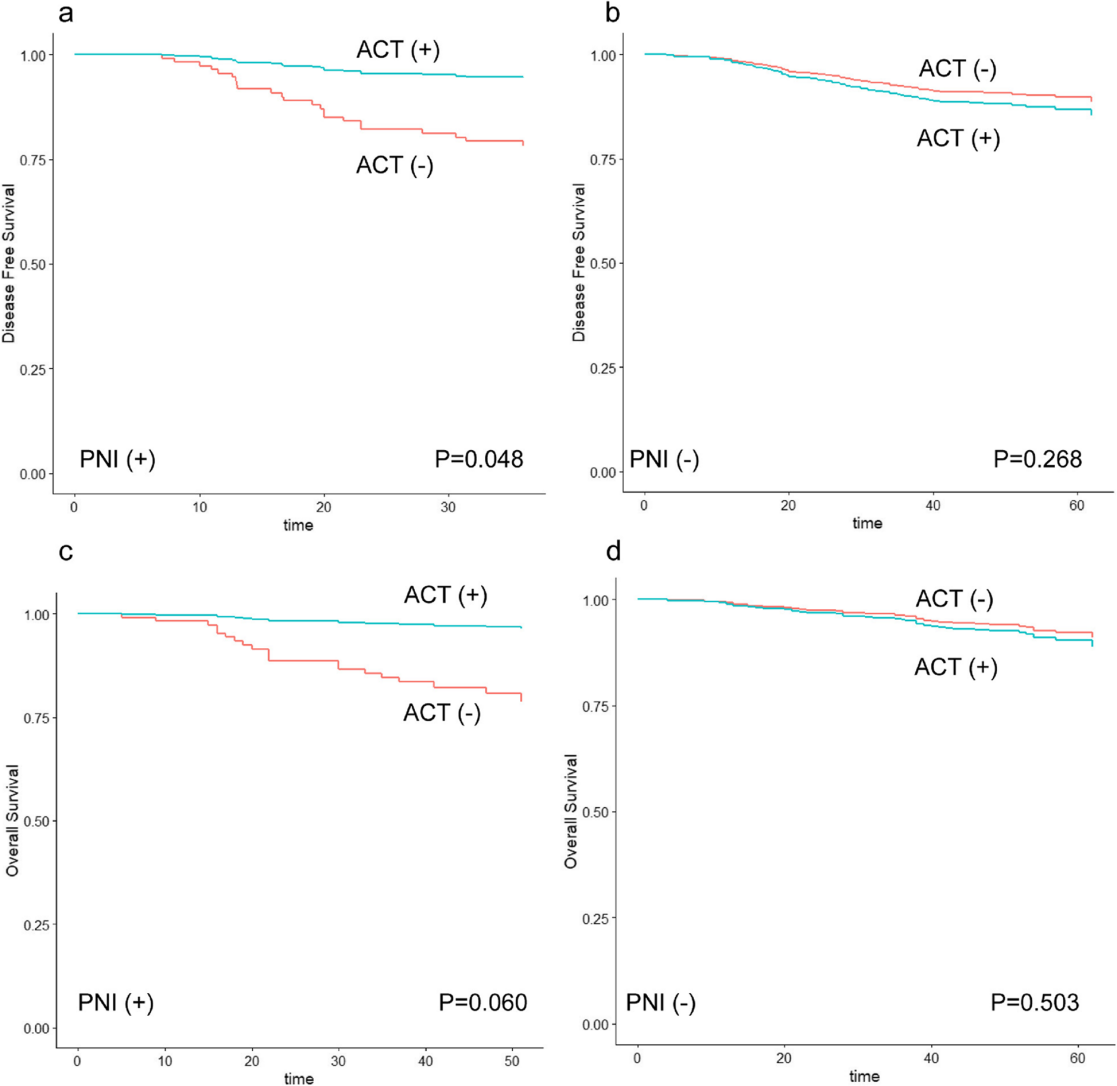
**a** Adjusted disease free survival curves calculated based on Cox model separately for subpopulations according to the use of ACT in PNI (+) patients. **b** Adjusted disease survival curves calculated based on Cox model separately for subpopulations according to the use of ACT in PNI (-) patients. **c** Adjusted overall survival curves calculated based on Cox model separately for subpopulations according to the use of ACT in PNI (+) patients. **d** Adjusted overall survival curves calculated based on Cox model separately for subpopulations according to the use of ACT in PNI (-) patients. ACT: Adjuvant chemotherapy; PNI: Perineural invasion

**Fig. 3:**
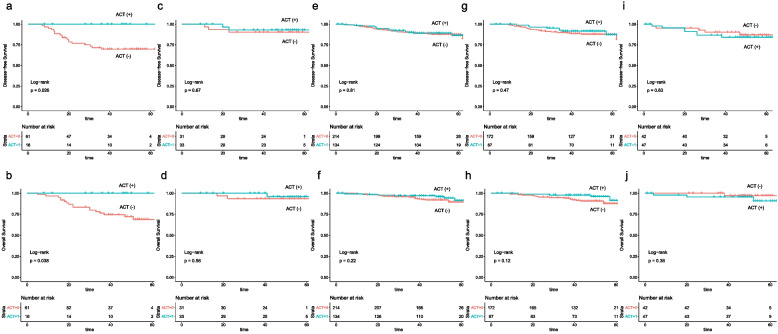
**a** Disease free survival curves calculated based on the Kaplan–Meier method separately for subpopulations according to the use of ACT in PNI (+) patients without other high-risk features. **b** Overall survival curves calculated based on the Kaplan–Meier method separately for subpopulations according to the use of ACT in PNI (+) patients without other high-risk features. **c** Disease free survival curves calculated based on the Kaplan–Meier method separately for subpopulations according to the use of ACT in PNI (+) patients with other high-risk features. **d** Overall survival curves calculated based on the Kaplan–Meier method separately for subpopulations according to the use of ACT in PNI (+) patients with other high-risk features. **e** Disease free survival curves calculated based on the Kaplan–Meier method separately for subpopulations according to the use of ACT in PNI (-) patients with other high-risk features. **f** Overall survival curves calculated based on the Kaplan–Meier method separately for subpopulations according to the use of ACT in PNI (-) patients with other high-risk features. **g** Disease free survival curves calculated based on the Kaplan–Meier method separately for subpopulations according to the use of ACT in PNI (-) patients with 1 high-risk feature. **h** Overall survival curves calculated based on the Kaplan–Meier method separately for subpopulations according to the use of ACT in PNI (-) patients with 1 high-risk feature. **i** Disease free survival curves calculated based on the Kaplan–Meier method separately for subpopulations according to the use of ACT in PNI (-) patients with ≥ 2 high-risk features. **j** Overall survival curves calculated based on the Kaplan–Meier method separately for subpopulations according to the use of ACT in PNI (-) patients with ≥ 2 high-risk features. ACT: Adjuvant chemotherapy; PNI: Perineural invasion

As age, ASA classification, and PNI were distributed unevenly between the ACT and non-ACT groups and were risk factors indicating poor survival, they were considered possible confounding factors of the chemotherapy effect on OS and were adjusted. Sex were also adjusted when considering the chemotherapy effect on DFS. We spotted a marginally significant ACT benefit on DFS and OS in patients with PNI (Fig. 2), however, no significant benefits were observed in other clinicopathological subgroups. The adjusted Cox proportional hazard models including an interaction term revealed significant multiplicative interaction between PNI and ACT regarding the hazard of DFS (HR 0.196, *P* = 0.038). Significant interaction between PNI and ACT in terms of disease-free survival was also supported by computing the interaction on an additive scale (RERI, − 1.996; 95%CI, -3.600 to -0.392) (Additional file 1: Table S1, Table 4). Similarly, significant multiplicative and additive interaction between PNI and ACT regarding the hazard of OS (HR for multiplicative interaction 0.112, *p* = 0.042; RERI, -2.842; 95%CI, -4.959 to -0.725) was also observed (Additional file 1: Table S2, Table 5). The phenomenon of ACT effect was consistent when considering the subpopulation of PNI(+) patients without other high-risk features, and no ACT effect was observed in the subpopulation of PNI(-) patients with other high-risk features (Fig. 3). Although no significant interaction could be observed regarding other clinicopathological factors, a trend of negative interaction which implies greater survival benefit from chemotherapy could be seen in patients with T4 tumors (DFS: HR for multiplicative interaction 0.766, *p* = 0.624; RERI, -0.423; 95%CI, -1.883 to 1.036; OS: HR for multiplicative interaction 0.609, *p* = 0.494; RERI, -0.484; 95%CI, -1.867 to 0.898) and < 12 lymph nodes examined (DFS: HR for multiplicative interaction 0.702, *p* = 0.749; RERI, -0.321; 95%CI, -2.113 to 1.472), while a trend of less benefit could be seen when patients were presenting with grade 3 histology (DFS: HR for multiplicative interaction 1.788, *p* = 0.266; RERI, 0.565; 95%CI, -0.446 to 1.577; OS: HR for multiplicative interaction 1.576, *p* = 0.514; RERI, 0.362; 95%CI, -0.588 to 1.311) and LVI (DFS: HR for multiplicative interaction 2.686, *p* = 0.193; RERI, 0.715; 95%CI, -0.368 to 1.799; OS: HR for multiplicative interaction 1.894, *p* = 0.513; RERI, 0.397; 95%CI, -0.635 to 1.429).

**Table 4:** Subgroup analysis and treatment-by-pathology interactions for DFS (adjusted for sex, age, ASA classification, PNI)

	**ACT VS Non-ACT** **HR** (95% CI)	**p**	**Multiplicative interaction**	**p for INT**^**M**^	**Additive interaction** **RERI** (95% CI)
Sex			1.452	0.507	0.391 (-0.729, 1.511)
Male	1.185 (0.671, 2.094)	0.559			
Female	0.592 (0.221, 1.584)	0.296			
Age			1.002	0.996	0.005 (-1.312, 1.322)
≥ 70	0.950 (0.402, 2.249)	0.908			
< 70	0.925 (0.510, 1.678)	0.798			
ASA			0.943	0.923	-0.118 (-2.502, 2.265)
III-IV	1.070 (0.367, 3.118)	0.902			
I-II	0.902 (0.520, 1.565)	0.715			
T4			0.766	0.624	-0.423 (-1.883, 1.036)
Yes	0.583 (0.227, 1.495)	0.261			
No	0.956 (0.520, 1.760)	0.886			
Lymph node harvest			0.702	0.749	-0.321 (-2.113, 1.472)
< 12	0.989 (0.116, 8.440)	0.993			
≥ 12	0.950 (0.575, 1.570)	0.841			
Grade			1.788	0.266	0.565 (-0.446, 1.577)
G3	1.561 (0.658, 3.701)	0.312			
G1-2	0.755 (0.408, 1.394)	0.369			
LVI			2.686	0.193	0.715 (-0.368, 1.799)
Yes	2.108 (0.496, 8.967)	0.313			
No	0.909 (0.521, 1.588)	0.738			
PNI			0.196	0.038	-1.996 (-3.600, -0.392)
Yes	0.227 (0.052, 0.987)	0.048			
No	1.347 (0.796, 2.280)	0.268			

*DFS* Disease-free survival, *ACT* Adjuvant chemotherapy, *HR* Hazardous ratio, *INT*^*M*^ Multiplicative interaction, *RERI* Relative excess risk due to interaction, *ASA* American Society of Anesthesiologists, *LVI* Lymphovascular invasion, *PNI* Perineural invasion

**Table 5:** Subgroup analysis and treatment-by-pathology interactions for OS (adjusted for age, ASA classification, PNI)

	**ACT VS Non-ACT** **HR** (95% CI)	**p**	**Multiplicative interaction**	**p for INT**^**M**^	**Additive interaction** **RERI** (95% CI)
Sex			5.848	0.101	0.936 (-0.038, 1.909)
Male	1.318 (0.668, 2.602)	0.426			
Female	0.142 (0.019, 1.078)	0.059			
Age			0.619	0.500	-1.015 (-3.145, 1.115)
≥ 70	0.563 (0.172, 1.844)	0.343			
< 70	0.914 (0.431, 1.941)	0.815			
ASA			0.798	0.783	-0.695 (-3.436, 2.046)
III-IV	0.689 (0.159, 2.988)	0.619			
I-II	0.812 (0.406, 1.624)	0.556			
T4			0.609	0.494	-0.484 (-1.867, 0.898)
Yes	0.344 (0.092, 1.280)	0.111			
No	0.939 (0.454, 1.945)	0.866			
Grade			1.576	0.514	0.362 (-0.588, 1.311)
G3	1.110 (0.328, 3.760)	0.866			
G1-2	0.714 (0.343, 1.483)	0.366			
LVI			1.894	0.513	0.397 (-0.635, 1.429)
Yes	1.561 (0.242, 10.066)	0.640			
No	0.821 (0.409, 1.649)	0.580			
PNI			0.112	0.042	-2.842 (-4.959, -0.725)
Yes	0.142 (0.019, 1.083)	0.060			
No	1.257 (0.643, 2.456)	0.503			

*OS* Overall survival, *ACT* Adjuvant chemotherapy, *HR* Hazardous ratio, *INT*^*M*^ Multiplicative interaction, *RERI* Relative excess risk due to interaction, *ASA* American Society of Anesthesiologists, *LVI* Lymphovascular invasion, *PNI* Perineural invasion

### Subpopulation Treatment Effect Pattern Plot (STEPP) analysis and sensitivity analysis

In Additional file 2: Fig. S1, the horizontal axis shows the median number of lymph node harvested and age within each category, with the number of patients in the corresponding subgroup displayed in parentheses. The difference in 3-year DFS and OS were shown on the vertical axis, with negative values favoring adjuvant treatment. The treatment benefit appeared to reverse at a cut-off point of 60 years old for DFS, but no similar reversal effect was detected in OS or in different lymph node harvest number subgroups. However, the multiplicative and additive interaction remained unsignificant even when altering the age cut-off point at 60 years old (Additional file 1: Table S3).

## Discussion

In this study, adverse pathological features in stage II colon cancer influenced the choice of whether to administer chemotherapy in clinical practice. In subgroup analysis, PNI had multiplicative interaction and additive interaction on ACT in terms of disease-free survival and overall survival. However, the presence of other adverse pathological features had no modification effect on the ACT, i.e., there may be no significantly different effect of chemotherapy in patients with/without these high-risk pathological features.

As NCCN guidelines has recommended patients presenting with high-risk factors for survival to receive ACT after curative surgery, previous research have attempted to prove the survival advantage of chemotherapy in patients with high-risk pathological features. According to a comprehensive study based on the Surveillance, Epidemiology, and End Results Program (SEER) database that included 65,831 individuals [7], ACT had adverse survival effect in 5-year cancer-specific survival (CSS) in stage II patients with high-risk pathology, no matter in patients with 1 high-risk factor (HR 1.407, 95% CI, 1.256–1.577) or with 2 or more risk factors (HR 1.305, 95% CI, 1.132–1.504). A study based on the NCDB database [8] and a prospective study in Japan [9], on the other hand, suggested that stage II colon cancer patients with high-risk pathology could benefit from ACT. The contradictory findings of these studies based on huge public datasets adjusted for confounding factors point to the necessity for more research into specific features, PNI, in particular.

Perineural invasion is the process of the infiltration of cancer cells inside or around nerves, and it is a well-established prognostic factor in a variety of tumors [15–17], including colon cancer. A systemic review involving 58 studies and 22,900 patients found that PNI is correlated with decreased 5-year DFS (HR 2.35, 95% CI, 1.97–3.08), CSS (HR 1.91, 95% CI, 1.50–2.42) and OS (HR 1.85, 95% CI, 1.63–2.12) in colorectal cancer [18]. Focusing on stage II colon cancer, Tu et al [19] demonstrated that PNI is an prognostic factor for CSS independent of T stage, age, tumor grade, etc. in 31805 stage II colon cancer patients from SEER database. The findings of our study led to a consistent conclusion, which showed a marginally significant prognostic effect of PNI on DFS and a significant effect on OS. However, the discussion of the response of ACT after curative surgery in stage II colon cancer patients depending on PNI status is relatively rare. Leijessen et al [20] analyzed the chemotherapy effect in PNI-positive node-negative patients, and found that neglecting ACT after surgery doubled the hazard of death, although without statistical significance. However, in order to demonstrate the predictive value of PNI on the response of chemotherapy and therefore to recommend chemotherapy in a certain risk group, two conditions need to be met: firstly, patients with high-risk pathological features (i.e. PNI) who received chemotherapy should have a significant improvement in survival (criterion 1); secondly, patients with high-risk pathological features (i.e. PNI) should have a greater improvement from ACT in survival than those without, indicating that the presence of an adverse pathological feature influences the prognostic effect of chemotherapy (criterion 2). Only the first question was addressed in Leijessen’s study, and researchers were also looking for an answer to the second question.

In 2016, Cienfuegos et al [21] discovered that in stage I-II PNI-positive colon cancer patients, receiving ACT significantly improved survival, however, no such difference were observed in PNI-negative patients. However, as stage I patients were not generally suggested to receive chemotherapy, and this study didn’t separate the analysis between stage I and II patients, possibly due to a lack of sample size (507 stage I-II colon cancer patients); What’s more, confounding factors were not adjusted when considering the ACT effect in this study. In contrary, in a study conducted by Tu et al [19] based on the SEER database including 57255 node negative patients, it is demonstrated that the receipt of ACT in T4 colon cancer is associated with decreased risk of cancer-specific mortality whether with or without PNI, and ACT was not a predictive factor for survival in T3N0M0 patients. Therefore, the authors concluded that PNI was not a predictive factor of response to adjuvant therapy. By utilizing a large public use database, this study was able to acquire a large sample size and account for potential confounding factors. However, DFS and OS status were not analyzed in this study, and the conclusion was insufficient since they reached their conclusions without comparing the ACT effect in patients with and without PNI quantitively. In our study, we attempted to bridge the gap by presenting the multiplicative as well as additive interaction of ACT and PNI in terms of DFS and OS, implying that the presence of PNI influenced the efficacy of ACT in patients with stage II colon cancer, and therefore it is the most prominent advantage of our study. We further testified our theory by suggesting that even for patients without any other high-risk features, PNI could indicate ACT effect, thus eliminating the possible confounding effect of other high-risk features; and in patients with high-risk features other than PNI, ACT showed no effect on survival, implicating that PNI is the most important feature suggesting ACT administration. However, the understanding of the mechanism of PNI on chemotherapy effect remains limited. But recent studies indicated that nerve-tumor interaction and neural regulation might be related to treatment sensitivity/resistance [22], whether the existence of PNI might influence the pathway relies on further investigation.

As for other high-risk pathological features, previous research has also attempted to prove the survival benefit of chemotherapy in patients with such features (criterion 1). Kumar et al [23] concluded that only patients with T4 tumor would have a better RFS, DSS, and OS from ACT, and Verhoeff et al [24] came to the same conclusion. In our study, patients with T4 tumors also showed a trend of improved survival from ACT. However, these studies neglected the chemotherapy effect in patients without these features. Therefore, we also dived into the difference of ACT benefit between these subgroups. Despite statistically significant interaction was only found apart in PNI and ACT, the findings concerning other pathological features were nonetheless intriguing. Patients with G3 histology, for example, have a trend toward decreased survival benefit, or even reversed, for those treated with ACT. Although these trends weren’t statistically significant, they’re still a hint for further consideration. It suggests that certain adverse pathological features may not lead to chemotherapy indication, or even be a sign of chemo-resistance instead. For example, previous studies have shown significant differences between mucinous adenocarcinoma and non-mucinous adenocarcinoma in terms of somatic mutation rates and copy number variation (CNV) in genes associated with resistance to 5-FU, oxaliplatin and irinotecan, which may imply a lower chemotherapy benefit in mucinous adenocarcinoma rather than non-mucinous adenocarcinoma [25–27].

Apart from the high-risk pathological factors mentioned in the NCCN guidelines, age was another important factor to consider when determining the administration of ACT. Past studies have preferred to use 70 as the age cut-off point, and large-scale analysis have found no survival benefit from adding oxaliplatin to patients above 70 years old [28, 29], which is why our study started with 70 as the age cut-off point. Based on STEPP analysis, we discovered a reversal of ACT effect for DFS at 60 years of age, though without statistical significance.

There are several limitations of this study. First is the inherent nature of recall bias in retrospective studies; secondly, the regimen and duration of chemotherapy varied in the ACT group, and we were unable to retrieve such information from patients in several centers in lack of in-time registration. Our study also has the following advantages, this study is a multi-center study of 8 tertiary hospitals in China, which increased the representativeness of this study; secondly, as a rarely used method, additive interaction and STEPP analysis could demonstrate the effect of pathological characteristics on the efficacy of ACT in a quantitive way.

Although PNI did show modification effect on ACT efficacy, we could ultimately reflect on the concept of whether all high-risk pathological indicates adjuvant chemotherapy. For inappropriate poor prognostic features, ACT may not serve to improve survival, but may instead add to psychological and financial burdens of patients, as well as to society. Apart from using pathological features such as PNI to predict ACT effect, genetic-based or immunology-based methods might also be applicable when it comes to the recommendation of treatment regimens [30, 31]. Fundamentally, we should look at the mechanism of why chemotherapy is beneficial or ineffective, not merely because it is a risk factor for prognosis.

## Conclusions

In conclusion, 931 stage II colon cancer patients were analyzed retrospectively in this study. According to our statistical analysis, PNI had prognostic value on survival outcomes. Additionally, significantly different effects of chemotherapy were observed in patients with/without PNI, indicating that patients with PNI could achieve greater survival benefit from ACT. No interaction was observed between other high-risk pathological features and ACT on survival benefit.

### Supplementary Information

**Additional file 1: Table S1. **Subgroup analysis and treatment-by-pathology interactions for DFS (unadjusted). Description: Significant interaction between PNI and ACT in terms of disease-free survival was supported by computing the interaction on multiplicative and additive scale (unadjusted) (HR for multiplicative interaction 0.166, p = 0.022; RERI, −2.258; 95%CI, -3.885, -0.631). **Table S2.** Subgroup analysis and treatment-by-pathology interactions for OS (unadjusted). Description: Significant interaction between PNI and ACT in terms of overall survival was supported by computing the interaction on multiplicative and additive scale (unadjusted) (HR for multiplicative interaction 0.104, p = 0.036; RERI, -3.139; 95%CI, -5.369, -0.908). **Table S3. **Subgroup analysis and treatment-by-age interactions for DFS (unadjusted and adjusted). Description: The treatment benefit appeared to reverse at a cut-off point of 60 years old for DFS, but no similar reversal effect was detected in OS or in different lymph node harvest number subgroups.

**Additional file 2: Figure S1. **STEPP analysis by age and lymph nodes harvest number. (a. STEPP analysis for age-DFS HR; b. STEPP analysis for age-OS HR; c. STEPP analysis for lymph node number-DFS HR; d. STEPP analysis for lymph node number-OS HR). Description: The multiplicative and additive interaction remained insignificant even when altering the age cut-off point at 60 years old.

## Data Availability

The datasets used and/or analyzed during the current study are available from the corresponding author on reasonable request.

## Abbreviations

DFS: Disease-free survival
OS: Overall survival
ACT: Adjuvant chemotherapy
RERI: Relative excess risk due to interaction
STEPP: The subpopulation treatment effect pattern plot
HR: Hazardous ratio
RFS: Relapse-free survival
NCCN: The National Comprehensive Cancer Network
LVI: Lymphovascular invasion
PNI: Perineural invasion
CRM: Circumferential resection margin
EPV: Events per variable
SD: Standard deviation
IQR: Interquartile range
ASA: American Society of Anesthesiologists
INT^M^: Multiplicative interaction
SEER: Surveillance, Epidemiology, and End Results Program
CSS: Cancer-specific survival
CNV: Copy number variation
