# Supplementary material for: Interaction analysis of high-risk pathological features on adjuvant chemotherapy survival benefit in stage II colon cancer patients: a multi-center, retrospective study
Source: BMC Cancer. 2023 Sep 18;23:797. doi: 10.1186/s12885-023-11196-4 (PMC10506231; doi:10.1186/s12885-023-11196-4)
Supplement: Supplementary file 1 — Additional file 1: Table S1. Subgroup analysis and treatment-by-pathology interactions for DFS (unadjusted). Description: Significant interaction between PNI and ACT in terms of disease-free survival was supported by computing the interaction on multiplicative and additive scale (unadjusted) (HR for multiplicative interaction 0.166, p = 0.022; RERI, −2.258; 95%CI, -3.885, -0.631). Table S2. Subgroup analysis and treatment-by-pathology interactions for OS (unadjusted). Description: Significant interaction between PNI and ACT in terms of overall survival was supported by computing the interaction on multiplicative and additive scale (unadjusted) (HR for multiplicative interaction 0.104, p = 0.036; RERI, -3.139; 95%CI, -5.369, -0.908). Table S3. Subgroup analysis and treatment-by-age interactions for DFS (unadjusted and adjusted). Description: The treatment benefit appeared to reverse at a cut-off point of 60 years old for DFS, but no similar reversal effect was detected in OS or in different lymph node harvest number subgroups. [file 12885_2023_11196_MOESM1_ESM.docx]

Table S1 Subgroup analysis and treatment-by-pathology interactions for DFS (unadjusted)

|  | **ACT VS Non-ACT**  **HR** (95% CI) | **P** | **Multiplicative**  **interaction HR** | **p for INT^M^** | **Additive interaction**  **RERI** (95% CI) |
| --- | --- | --- | --- | --- | --- |
| Sex  Male  Female | 0.848 (0.490, 1.468)  0.574 (0.221, 1.491) | 0.556  0.255 | 1.471 | 0.492 | 0.227 (-0.632, 1.086) |
| Age  ≥70  <70 | 1.000 (0.425, 2.354)  0.947 (0.526, 1.707) | 1.000  0.856 | 1.062 | 0.910 | 0.065 (-2.100, 2.229) |
| ASA  III-IV  I-II | 0.938 (0.324, 2.712)  0.850 (0.499, 1.449) | 0.905  0.551 | 1.120 | 0.851 | 0.009 (-2.959, 2.977) |
| T4  Yes  No | 0.507 (0.210, 1.226)  0.722 (0.399, 1.308) | 0.132  0.282 | 0.704 | 0.517 | -0.792 (-2.376, 0.791) |
| Lymph node harvest  <12  ≥12 | 0.677 (0.082, 5.625)  0.785 (0.483, 1.277) | 0.718  0.329 | 0.836 | 0.871 | -0.189 (-2.020, 1.642) |
| Grade  G3  G1-2 | 1.136 (0.497, 2.596)  0.663 (0.365, 1.204) | 0.763  0.177 | 1.703 | 0.306 | 0.436 (-0.354, 1.226) |
| LVI  Yes  No | 1.769 (0.442, 7.073)  0.701 (0.409, 1.202) | 0.420  0.197 | 2.462 | 0.235 | 0.688 (-0.331, 1.707) |
| PNI  Yes  No | 0.177 (0.041, 0.753)  1.039 (0.625, 1.730) | 0.019  0.882 | 0.166 | 0.022 | -2.258 (-3.885, -0.631) |

DFS: Disease-free survival; ACT: Adjuvant chemotherapy; HR: Hazardous ratio; INT^M^: Multiplicative interaction; RERI: relative excess risk due to interaction; ASA: American Society of Anesthesiologists; LVI: Lymphovascular invasion; PNI: Perineural invasion.

Table S2 Subgroup analysis and treatment-by-pathology interactions for OS (unadjusted)

|  | **ACT VS Non-ACT**  **HR** (95% CI) | **P** | **Multiplicative**  **interaction HR** | **p for INT^M^** | **Additive interaction**  **RERI** (95% CI) |
| --- | --- | --- | --- | --- | --- |
| Sex  Male  Female | 0.851 (0.443, 1.636)  0.144 (0.019, 1.071) | 0.629  0.058 | 5.951 | 0.097 | 0.692 (-0.017, 1.401) |
| Age  ≥70  <70 | 0.615 (0.189, 1.999)  0.926 (0.441, 1.946) | 0.419  0.840 | 0.666 | 0.567 | -1.155 (-3.851, 1.541) |
| ASA  III-IV  I-II | 0.638 (0.148, 2.746)  0.733 (0.375, 1.435) | 0.546  0.365 | 0.867 | 0.861 | -0.993 (-4.500, 2.514) |
| T4  Yes  No | 0.347 (0.101, 1.191)  0.662 (0.326, 1.345) | 0.093  0.254 | 0.537 | 0.391 | -0.808 (-2.407, 0.791) |
| Grade  G3  G1-2 | 0.764 (0.240, 2.437)  0.604 (0.295, 1.231) | 0.650  0.165 | 1.251 | 0.747 | 0.236 (-0.541, 1.012) |
| LVI  Yes  No | 1.218 (0.203, 7.302)  0.594 (0.303, 1.164) | 0.829  0.129 | 2.114 | 0.443 | 0.529 (-0.503, 1.562) |
| PNI  Yes  No | 0.099 (0.013, 0.737)  0.915 (0.479, 1.747) | 0.024  0.787 | 0.104 | 0.036 | -3.139 (-5.369, -0.908) |

OS: Overall survival; ACT: Adjuvant chemotherapy; HR: Hazardous ratio; INT^M^: Multiplicative interaction; RERI: relative excess risk due to interaction; ASA: American Society of Anesthesiologists; LVI: Lymphovascular invasion; PNI: Perineural invasion.

Table S3 Subgroup analysis and treatment-by-age interactions for DFS (unadjusted and adjusted)

|  | **ACT VS Non-ACT**  **HR** | **p** | **Multiplicative interaction HR** | **p for INT^M^** | **Additive interaction**  **RERI** |
| --- | --- | --- | --- | --- | --- |
| **DFS** |  |  |  |  |  |
| Age (unadjusted)  ≥60  <60 | 0.984 (0.551, 1.757)  0.793 (0.339, 1.853) | 0.956  0.592 | 1.237 | 0.686 | 0.172 (-1.141, 1.484) |
| Age (adjusted)  ≥60  <60 | 0.961 (0.537, 1.720)  0.815 (0.346, 1.921) | 0.894  0.640 | 1.200 | 0.728 | 0.141 (-1.028, 1.310) |
| **OS** |  |  |  |  |  |
| Age (unadjusted)  ≥60  <60 | 0.683 (0.324, 1.439)  1.143 (0.363, 3.603) | 0.315  0.819 | 0.591 | 0.451 | -1.350 (-4.222, 1.522) |
| Age (adjusted)  ≥60  <60 | 0.667 (0.316, 1.411)  1.279 (0.394, 4.152) | 0.289  0.682 | 0.582 | 0.438 | -1.171 (-3.722, 1.380) |

DFS: Disease-free survival; OS: Overall survival; ACT: Adjuvant chemotherapy; HR: Hazardous ratio; INT^M^: Multiplicative interaction; RERI: relative excess risk due to interaction; ASA: American Society of Anesthesiologists; LVI: Lymphovascular invasion; PNI: Perineural invasion.
