# Supplementary material for: Interaction analysis of high-risk pathological features on adjuvant chemotherapy survival benefit in stage II colon cancer patients: a multi-center, retrospective study
Source: BMC Cancer. 2023 Sep 18;23:797. doi: 10.1186/s12885-023-11196-4 (PMC10506231; doi:10.1186/s12885-023-11196-4)
Supplement: Supplementary file 2 — Additional file 2: Figure S1. STEPP analysis by age and lymph nodes harvest number. (a. STEPP analysis for age-DFS HR; b. STEPP analysis for age-OS HR; c. STEPP analysis for lymph node number-DFS HR; d. STEPP analysis for lymph node number-OS HR). Description: The multiplicative and additive interaction remained insignificant even when altering the age cut-off point at 60 years old. [file 12885_2023_11196_MOESM2_ESM.docx]

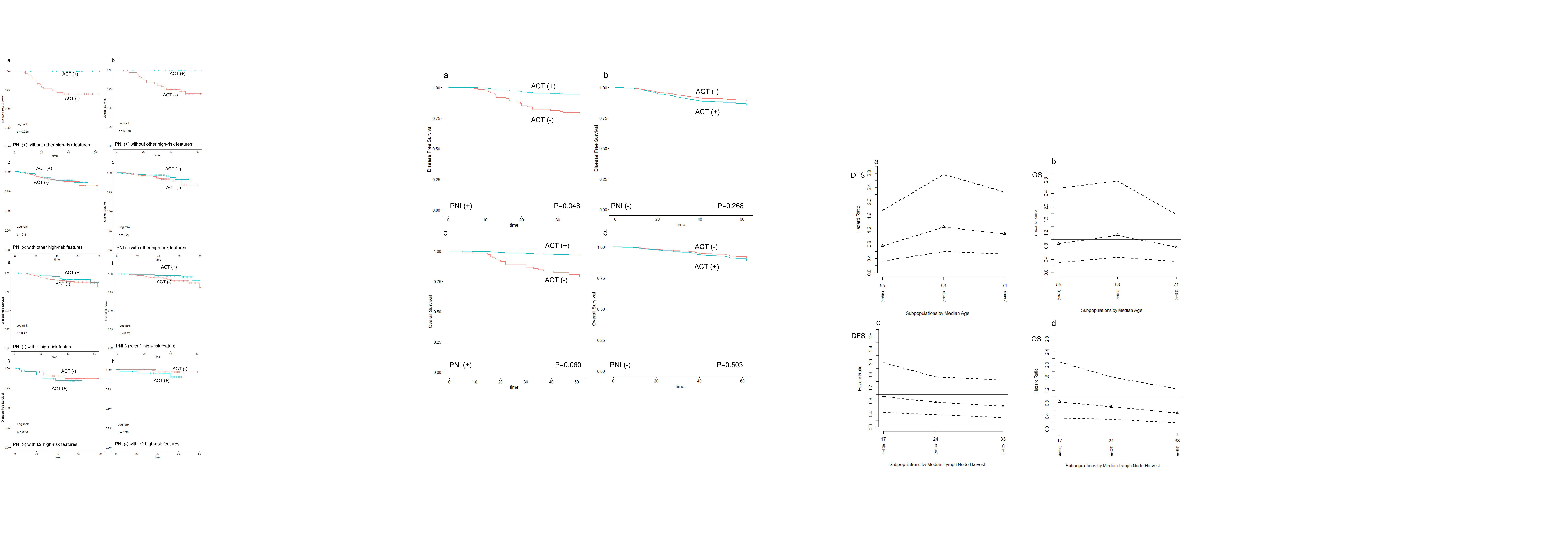


Figure S1 STEPP analysis by age and lymph nodes harvest number (a. STEPP analysis for age-DFS HR; b. STEPP analysis for age-OS HR; c. STEPP analysis for lymph node number-DFS HR; d. STEPP analysis for lymph node number-OS HR). STEPP: The subpopulation treatment effect pattern plot; DFS: Disease-free survival; OS: Overall survival; HR: Hazardous ratio.
